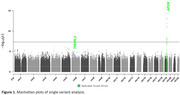# Peruvian Population Empowers Discovery of a *TREM2* Missense Variant in Alzheimer Disease

**DOI:** 10.1002/alz70855_105970

**Published:** 2025-12-24

**Authors:** Bilcag Akgun, Mario Cornejo‐Olivas, Pedro R Mena, Larry D Adams, Katrina Celis, Patrice G Whitehead, Kara L Hamilton‐Nelson, Julia Rios‐Pinto, Angel Medina‐Colque, Clifton L. Dalgard, Eden R. Martin, Ivan Cornejo‐Herrera, Maryenela Illanes‐Manrique, Edward Ochoa‐Valle, Elison Sarapura‐Castro, Koni Mejia ‐ Rojas, Victoria Marca‐Ysabel, Sheila Castro‐Suarez, Anthony J Griswold, Rosario Isasi, Katalina F. McInerney, Michael L Cuccaro, Jeffery M Vance, Farid Rajabli, Margaret Pericak‐Vance, Alzheimer's Disease Sequencing Project (ADSP)

**Affiliations:** ^1^ John P. Hussman Institute for Human Genomics, University of Miami Miller School of Medicine, Miami, FL, USA; ^2^ Neurogenetics Working Group, Universidad Cientifica del Sur, Lima, Peru; ^3^ Neurogenetics Research Center, Instituto Nacional de Ciencias Neurológicas, Lima, Peru; ^4^ University of Miami Miller School of Medicine, Miami, FL, USA; ^5^ Universidad Peruana Los Andes, Huancayo, Peru; ^6^ Dirección Regional de Salud de Puno, Puno, Peru; ^7^ Department of Anatomy, Physiology, and Genetics, Uniformed Services University of the Health Sciences, Bethesda, MD, USA; ^8^ Dr. John T. Macdonald Foundation Department of Human Genetics, University of Miami Miller School of Medicine, Miami, FL, USA; ^9^ Hospital Hipólito Unanue de Tacna, Tacna, Peru; ^10^ Atlantic Fellow for Equity in Brain Health at Global Brain Health Institute (GBHI), San Francisco, CA, USA; ^11^ Hospital Regional de Cusco, Cusco, Peru; ^12^ EDMECON Continuing Medical Education, Lima, Peru; ^13^ Daniel Alcides Carrion National Hospital, Callao, Peru; ^14^ CBI en Demencias y Enfermedades Desmielinizantes del Sistema Nervioso, Instituto Nacional de Ciencias Neurológicas, Lima, Peru; ^15^ Department of Neurology, University of Miami Miller School of Medicine, Miami, FL, USA

## Abstract

**Background:**

Increasing ethnic/ancestral diversity in genetic studies is critical for defining the genetic architecture of Alzheimer disease (AD) and maximizing the opportunities for identifying genetic protective and risk alleles. The Peruvian population, with up to ∼80% of Amerindian ancestry, provides a unique opportunity to leverage Amerindian ancestry for deeper insights into AD mechanism. We performed the genome‐wide association study (GWAS) in the Peruvian population to characterize known AD genetic risk loci.

**Method:**

567 individuals (215 AD; 352 cognitively unimpaired) were included in these analyses. We performed GWAS on the Whole Genome Sequencing (WGS) dataset using a generalized linear mixed model, adjusting for sex, age, and population substructure as fixed effects and the genetic relationship matrix as a random effect. For follow‐up analysis, we extracted all nominal significant known AD variants and calculated linkage disequilibrium (LD) with surrounding SNPs. Variants with an R^2^ ≥ 0.8 with the index SNP were annotated and further evaluated. To assess whether the associations were ancestry‐specific, we calculated the local ancestry of the regions corresponding to these variants.

**Result:**

We replicated two known AD loci *APOE4* allele and *TREML2* marker (rs60755019). Fine‐mapping analysis at the *TREML2* loci identified a *TREM2* missense exonic marker (p.H157Y, OR=3.3 [1.9‐5.9], *p* = 4x10^‐5^, CADD=23.2) having strong LD with the *TREML2* risk marker in Peruvians. LA analysis showed that p.H157Y is located on an Amerindian ancestral background. p.H157Y is extremely rare across frequency databases (gnomAD, HGDP, and 1000 Genomes) except in Admixed American population ∼0.02. Additionally, *APOE* showed a strong association with AD, with an effect size (OR=4.3 [3.0‐6.1], *p* = 4x10^‐15^) higher that was observed in non‐Hispanic White populations.

**Conclusion:**

Peruvian GWAS identified Amerindian ancestry specific exonic AD susceptibility variant p.H157Y. This variant has been previously reported as AD marker in Han Chinese populations with extreme effect size, but its rarity and contradictory findings in other groups suggest a potential ancestry‐specific effect. Functional studies indicate that it may enhance *TREM2* shedding, impacting microglial function and contributing to AD pathogenesis. These observations highlight the power of examining diverse populations to gain a more comprehensive view of the genetic architecture of AD.